# School-based vision screening in Quetta, Pakistan: a qualitative study of experiences of teachers and eye care providers

**DOI:** 10.1186/s12889-021-10404-9

**Published:** 2021-02-16

**Authors:** Stevens Bechange, Munazza Gillani, Emma Jolley, Robina Iqbal, Leena Ahmed, Muhammed Bilal, Itfaq Khaliq Khan, Sumrana Yasmin, Elena Schmidt

**Affiliations:** 1 Sightsavers Pakistan Country Office, Plot 3-A, Street 7, Sector G-10/2, Islamabad, Pakistan; 2 Sightsavers - United Kingdom, 35 Perrymount Road, Haywards Heath, West Sussex, RH16 3BW UK; 3 Rawalpindi, Pakistan

**Keywords:** Task shifting, Teachers, Vision screening, Children, School health, Visual impairment, South Asia, Qualitative, Pakistan

## Abstract

**Background:**

Visual impairment in children is a significant public health problem affecting millions of children globally. Many eye problems experienced by children can be easily diagnosed and treated. We conducted a qualitative study with teachers and optometrists involved in a school-based vision screening programme in Quetta district of Pakistan to explore their experiences of training, vision screening and referrals and to identify factors impacting on the effectiveness of the programme.

**Methods:**

Between April 2018 and June 2018, we conducted semi-structured in-depth interviews with 14 teachers from eight purposefully selected schools with high rates of inaccurate (false positive) referrals. Interviews were also conducted with three optometrists from a not-for profit private eye care hospital that had trained the teachers. Interviews were audio recorded and professionally transcribed. NVIVO software version 12 was used to code and thematically analyze the data.

**Results:**

Findings suggest that the importance of school-based vision screening was well understood and appreciated by the teachers and optometrists. Most participants felt that there was a strong level of support for the vision screening programme within the participating schools. However, there were a number of operational issues undermining the quality of screening. Eight teachers felt that the duration of the training was insufficient; the training was rushed; six teachers said that the procedures were not sufficiently explained, and the teachers had no time to practice. The screening protocol was not always followed by the teachers. Additionally, many teachers reported being overburdened with other work, which affected both their levels of participation in the training and the time they spent on the screening.

**Conclusions:**

School-based vision screening by teachers is a cost-effective strategy to detect and treat children’s vision impairment early on. In the programme reviewed here however, a significant number of teachers over referred children to ophthalmic services, overwhelming their capacity and undermining the efficiency of the approach. To maximise the effectiveness and efficiency of school-based screening, future initiatives should give sufficient attention to the duration of the teacher training, experience of trainers, support supervision, refresher trainings, regular use of the screening guidelines, and the workload and motivation of those trained.

**Supplementary Information:**

The online version contains supplementary material available at 10.1186/s12889-021-10404-9.

## Background

Visual impairment in children is a significant public health problem affecting millions of children globally, and at least 12.8 million children are visually impaired due to uncorrected refractive errors (URE) [1]. Visual impairment in childhood hinders educational attainment and development and subsequently future career opportunities and socio-economic wellbeing [2, 3]. Also, children with unattended eye health problems have an increased risk of visual impairment or loss of vision in adulthood [4, 5]. In addition to eye diseases associated with poor vision in one or both eyes, children can frequently experience conditions, which do not cause loss of vision but impact on their daily activities, social interactions and learning, including infectious and non-infectious conjunctivitis and allergies [6]. Many eye problems experienced by children can be easily diagnosed and treated with eye drops or optical devices, such as spectacles or lenses [7]. Yet, the majority of children living in resource-poor settings do not have access to eye care services, which impacts negatively on their quality of life and education [8].

One of the main barriers to accessing eye care services in many low- and middle-income countries (LMICs) is the limited number and unequal distribution of eye care providers, who tend to be located in large urban centres and therefore inaccessible for a large proportion of the population, particularly those in rural and remote areas [9, 10]. Families in such locations often cannot take their children to health facilities for regular vision check-ups due to time and transport constraints [11]. For this reason, school-based eye health programmes have been promoted as critical interventions to detect and address eye health problems in children earlier. The intervention is based on task-shifting where teachers are trained to conduct vision screening of all children in the school or specific age groups. Those who fail the screening are examined by qualified eye care practitioners, often optometrists, who either receive referrals in their eye care clinics or visit schools. These practitioners then provide treatment or optical devices to children they can treat and refer more complex cases to higher level facilities [12].

Task shifting from health to non-health professionals has been implemented in many countries experiencing health workforce shortages as a mechanism to meet the rising demand for health services [13, 14]. It is a form of service delegation where tasks that are traditionally performed by qualified health care workers are identified and assigned to individuals with fewer or non-health qualifications but with the ability to learn the skills required to perform the desired tasks. Training teachers to perform some less technical health care delivery tasks within the school environment has been found not only to relieve health professionals of unwarranted workload but also to expand the total output of education and primary health care systems in resource-limited settings [7, 15–17]. Various global initiatives acknowledge the critical role of schools as a strategic platform for programmatic health interventions [18, 19]. In many countries, tasks such as screening for malnutrition, immunizations and deworming have been successfully performed by trained teachers in the school setting [20, 21].

Using teachers to conduct visual screening has shown several advantages in terms of access, cost–effectiveness and equity of paediatric eye care [22–24]. However, in order for screening programmes to be effective, it is important to minimise errors which may include both false negatives, where children with abnormal vision are identified as normal and do not receive the required referral; and false positives, where children with normal vision are identified as abnormal and unnecessarily referred to specialist services. Available studies show that while teachers have demonstrated adequate accuracy in screening in a variety of settings, sensitivity was lower with younger children [25], lower (< 6/18 vision level) screening cut-off thresholds for referral [22, 26] and specific types of vision chart (e.g. regular Snellen alphabet and Tumble 14 ‘E’ charts which are more time consuming) [26]^.^ The effectiveness of screening programmes has also been shown to be dependent on teacher motivation, confidence, awareness of students, examination location and tools [23]. However, the number of studies exploring experiences of those involved in school-based vision screening programmes continues to be limited.

Against this background, we conducted a qualitative study with teachers and ophthalmic staff involved in a vision screening programme in Quetta district of Baluchistan Province of Pakistan. The study aimed to explore programme participants’ experiences of training, vision screening and referrals in order to identify factors impacting on teachers’ performance and subsequently the effectiveness of the programme. In this study we focused specifically on the schools, where teachers showed a high degree of inaccurate referrals by identifying a considerable number of false positives (69–96%) resulting in the overburden of local eye care providers and sub optimal efficiency in service delivery.

## Methods

### Setting and context

The study was integrated within the ‘Giving vision to future visionaries’ programme implemented by Sightsavers and its partners’ in Quetta district of Baluchistan Province, Pakistan between March 2016 and February 2018. The province has a population of 12.3 million people with an estimated 2 million children aged below 15 years [27]. The programme focused on training teachers to screen children within the school premises for eye and vision problems and referring those identified as having issues to nearby eye health facilities. Teachers were not renumerated for the screening work. Both government and private schools were included. Private sector schools in Pakistan generally follow the same guidelines from the education department as government schools; so, there were no major differences in how the vision screening programme was delivered in the public and private sector schools.

**Fig. 1:**
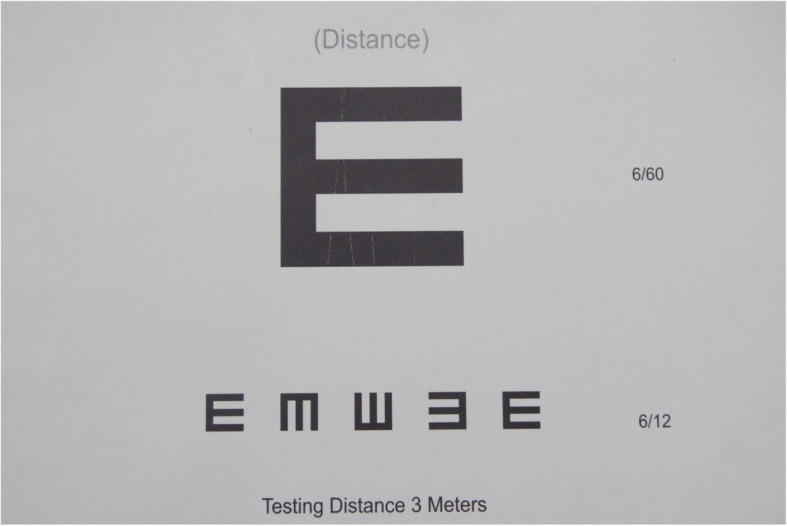
Vision screening chart

The teachers were trained by optometrists in how to screen children and record their findings, as well as giving them a basic understanding of primary eye health conditions that affect children. The training content included: common eye diseases; signs of health eye; vision screening (distance and near); referral management and eye health messages. The vision screening chart (Fig. 1) used had high contrast black on white, with a dark surround which improves reliability. One side was used for distance vision screening and the other was meant for near vision. Teachers were trained in near vision screening, but the trainers told them not to conduct it and indeed the referral slip they were given only had space for distance vision screening. The trainers told the teachers that they were not to conduct near vision screening because the programme prioritised distance vision screening due to resources constraints. The visual acuity cut-off of distance vision screening was recognising four of the five optotypes at 6/12 level at a distance of 3 m. Children were tested with their spectacles if they already had vision correction. Each eye had to be tested individually at the appropriate test distance (3 m). Failure of distance vision screening was defined as unable to correctly identify 4 of the 5 optotypes in any eye.

**Fig. 2:**
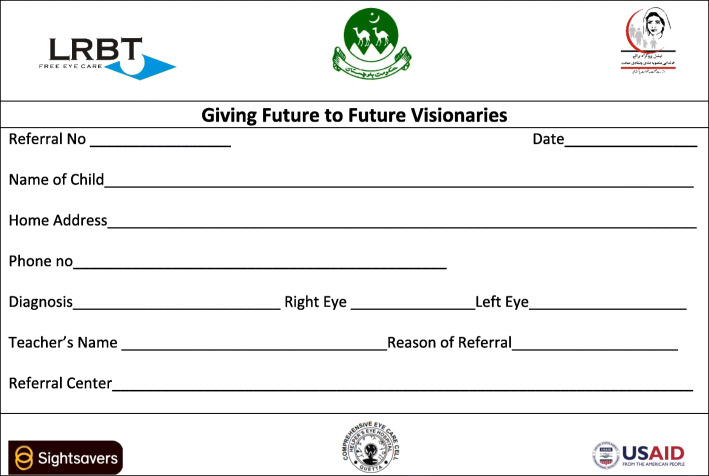
Referral slip

A total of 845 schools were included in the programme. Fifteen teachers per school were trained to screen all school children aged 5 years and older. Three fulltime trainers were recruited to the programme. School children, who failed the screening were issued a referral slip (Fig. 2) to be presented at the local community eye clinic for further examination and treatment. Teachers were also instructed to refer children for further eye examination if they had one or more of the signs of unhealthy eyes: the cornea is not transparent, the pupil is not round and black, one eye turns inwards or outwards (strabismus), the eye(s) are red with discharge (conjunctivitis or allergy), there is a foamy white spot or a dry wrinkled patch on the bulbar conjunctiva. The screening was conducted once in each school during the programme. All eye care services were provided by the same not-for profit private eye care provider through a network of their vision centres and community eye clinics. Over 2 years, 100,846 children from 429 primary schools were screened by the teachers. However, we could not establish the overall referral and false positive rates for the entire programme over the 2 years, as a large proportion of referral records were either never kept or had been lost by the time of the study.

In early 2017, routine analysis of programme data had revealed a remarkably high rate of false positives, ranging from 73 to 96%. The programme staff got concerned that the high number of false positives were increasing the workload of the ophthalmic staff and the costs of the programme and was negatively affecting the trust of the programme by the parents. The study presented here was developed in response to these concerns.

### Study design and sampling

This was a cross-sectional qualitative study that used face-to-face semi-structured interviews to collect data. We used a purposive, non-probabilistic sampling to select eight schools with the highest recorded rates of false positives, including six public and two private schools. The rate of false positives in the included schools ranged from 69 to 96% (Table 1). The programme did not record referrals for individual teachers. The selection of teachers for the interview took place on the day of the interviews based on the teachers’ availability and to ensure the representation of both men and women. In total 14 teachers were selected and agreed to be interviewed (Table 2). In addition, three optometrists involved in the teacher training and children examination were purposively selected and interviewed.

**Table 1:** Purposively selected schools for the study

School	Category of school	Total children screened	Children referred by teachers	Referral rate (%)	Optometrist confirmed cases	False positive (%)
Sch1	Public	117	54	46	2	96
Sch2	Public	473	184	39	9	95
Sch3	Private	1583	631	40	75	88
Sch4	Public	465	223	48	28	87
Sch5^a^	Public	764	207	27	50	76
Sch6	Public	1029	203	20	52	74
Sch7	Private	105	60	57	17	72
Sch8	Public	643	145	23	45	69

^a^Government School for Children with Special needs (Boys)

**Table 2:** Distribution of the 14 teachers across eight schools

School	Total number of teachers	Interviewed teachers		Total number of teachers interviewed
		Male	Female	
Sch1	14	1	1	2
Sch2	31	1	0	1
Sch3	31	0	2	2
Sch4	5	0	2	2
Sch5	20	1	0	1
Sch6	36	0	2	2
Sch7	27	2	0	2
Sch8	11	0	2	2

### Data collection

Data were collected between April 01 and June 30, 2018 by two trained interviewers with a qualitative research background (one female, one male). The interviewers had no previous relationships with the programme or the interviewees. The interviews followed a semi-structured topic guide and were conducted face-to-face in Urdu. The interviews were conducted in private locations at the participants’ place of work and lasted between 30 and 44 min. All interviews were audio-recorded. Topic guides were developed for this study based on the study objectives. The topic guides were pilot tested for question clarity with three volunteers (two teachers and one optometrist) and modified as new lines of inquiry were identified during the interviews. The topic guide for the teachers (Supplementary file_1) explored their experience of vision screening training, their interest and motivation to do the screening, the actual process of conducting it, the use of guidelines and referral forms, any challenges experienced, and any support received. The topic guide for the optometrists (Supplementary file_2) covered their experience of providing training, supporting teachers’ in schools and managing the referred cases. All questions were open-ended, and the interviewer had minimum interference in the interview, letting participants share their feelings and experiences.

### Data management and analysis

Audio recordings were professionally transcribed verbatim and translated into English. The translations were verified against the transcripts by two bilingual members of the team (IK, LA). The data were coded and analysed thematically using QSR NVIVO software version 12. The coding of the data was completed by a team of three researchers (RI, IK, SB) who were not involved in the fieldwork. The coding framework was developed using the first three transcripts and discussed by the data analysis team. Additional codes were added during the analysis of the next four transcripts. The final framework was agreed after coding seven transcripts. This framework was reapplied to the previously coded seven transcripts and the remaining ten transcripts by the three researchers (RI, IK, SB) independently and compared their coding and interpretations. Each subsample of participants’ transcripts was coded as a group, with teachers and optometrists treated as two separate groups. The codes were reviewed and organised into themes and sub-themes during a two-day meeting of the analysis team in Islamabad. Findings were shared with the programme staff for their validation and feedback.

### Ethical considerations

The study was approved by the Research Ethical Committee (REC) of Layton Rahmatulla Benevolent Trust (LRBT) (ref. CO/OPS3/1752). Additional permissions were obtained from all relevant local government authorities and head teachers of the participating schools. Participation was voluntary and there was no financial compensation for those participating in the study. All participants were given information about the study and the use of data; and all participants provided written informed consent. Participants were given assurance of confidentiality and strict protection of the collected data. Transcripts were anonymized using participant codes and stored on a secure computer at Sightsavers Pakistan Country Office.

## Results

Of the fourteen teachers who participated in the study, nine were male and five were female. Four taught in private schools, and the rest were in government schools, including one school for children with special needs. The mean number of years of teaching experience was 4.4. years (SD = 2.1). All three ophthalmic staff were male and worked as optometrists.

Analysis generated six overarching themes. These themes, with illustrative quotes, are presented below.

### Teacher training

The training for teachers was carried out in small groups within each school, and all sessions were gender segregated to conform with the cultural context. The training covered a range of topics, including setting up the screening environment, the use of the screening chart, the distance between the chart and the student for near vision (although this was not performed) and distance vision tests, the instructions given to the student, recording the result and completing a referral form, if needed. A pre- and post- training assessment was carried out to assess teachers’ knowledge and skills. The study did not have access to the results of the assessments, but the optometrists interviewed reported improvements in the teachers’ assessment scores following the training.

The opinions about the quality of the training among the teachers were mixed. Some teachers described it as particularly good and effective, whereas others were not fully satisfied. Eight teachers felt that the content of the training required more time and further discussions were needed to better understand the concepts and procedures. In fact, the duration of the training was one of the key issues highlighted in the interviews with both the trainers and the trainees. The training was scheduled to take place over 5 hours, between 0900 am and 2.00 pm. However, the duration was reportedly reduced to between one and two and a half hours, largely because the optometrists were expected to train three schools per day but there was only one vehicle to drop them off and pick them up from the schools. The coordination of the timing was difficult, particularly, when there were additional contextual challenges, such as extreme weather conditions or public holidays and festivals, as one optometrist explained:

“First of all, we have only one vehicle to move. Since we have to meet the target, so we go to three places in a day and the same vehicle is used to drop one person and pick the other one, so that creates a complication and challenge. Sometimes harsh weather is a challenge and another one is the holidays … like … Eid and other festivals” [**Optometrist 2].**

While all the trainers reported that the training covered only the basics and the timing was sufficient, the teachers found the training too short to cover all the topics they were required to learn. The teachers wanted more time to better understand the screening procedures, ask questions and practice the skills:

“In this case, I am not satisfied because the training session was too short. I think it should be a three-day training session. The training was hardly for sixty to ninety minutes. So, we were not very clear.” [**Female teacher, government school]**

Whereas the three optometrists interviewed were the trainers of the teachers interviewed, there was a discrepancy in the views of the optometrists and the teachers on the practical sessions included in the training. All the optometrists reported that the teachers had an opportunity to practice screening in small groups; half of the teachers observed that there were no practical sessions in the presence of the trainer.

There were also difficulties in the recollection of the training on distinct types of screening. The teachers were trained on conducting both the near vision test and the distance vision test but advised not to perform near vision test. The vision screener given to the teachers had two sides for the two tests. For most teachers, this appears to have created confusion. One teacher, for example, who did not remember the distinction between near and distance vision tests, said that she was not sure which side of the chart they should use:

“We have to check both near vision and distance vision, but the trainers who gave us the training earlier did not teach us how to check near and distance vision. They only said that you will use the E chart card from the 3 metres distance.” [**Female teacher, private school]**

All the three optometrists were generally positive about the training. They praised the participation of the teachers, especially the older teachers. However, in a number of schools, they felt that the teachers did not take the training seriously. There was no refresher training scheduled as part of the project.

### Testing kits and materials

Following the training, the teachers were provided with a testing kit, which included an E chart card, a measuring tape, a screening guide, and a leaflet with eye health education messages (Supplementary file_3). All teachers reported having and using these materials. Eleven teachers could recollect the purpose of the E chart, the measuring tape, and the screening guide and why these were important for accurate testing. However, most teachers reported using the E chart card and the measuring tape only. The guidelines in most cases were placed in a school cupboard and never used. This was surprising as the guidelines were only a two-page document which teachers could have easily pasted on the wall where screening was set up. Several teachers did not know where they were stored.

“No, it [the guidelines] was in the classroom. I do not know what the kids did to it. I don’t have it with me now.” [**Female teacher, government school]**

“These materials may be present in our school with our madam [head teacher], but I do not have them**.” [Male teacher, government school]**

### Screening in practice

Most teachers (13/14) reported that they knew that the screening must be carried out in a well-lit and quiet environment. Of these, 12 said that they had carried out the screening in large halls, libraries, and verandas. In some schools, it was conducted in the classrooms with sufficient light. Eleven teachers were confident that they had acquired the skills to carry out the screening and reported that they followed the procedures they had learnt during the training. Six teachers however could not clearly recall what the procedure was; three were not sure about the exact distance between the student and the E chart; four reported incorrect distances.

One of the key issues highlighted during the interviews was incorrect use of the E chart by the teachers. Although the teachers were taught to rotate the card holding the corners, none of the teachers interviewed reported doing it. Some teachers placed the chart on a board, some gave it to a student to hold. The optometrists interviewed were aware of this issue and felt that the incorrect use of the E chart was a leading contributor to the inaccurate screening:

“ … the issue is with the use of the E card. We asked the teachers to move it … , but the teachers usually fix it against the wall or ask the children to hold it. It means they did not move it … to read the E card from all directions”. [**Optometrist 3**]

Furthermore, although ten teachers carried out vision screening themselves, four teachers (all from government schools) reported being too busy with other commitments. They delegated screening to the students; of these, three said that the process of screening by a team of students was more manageable in term time; they also argued that the students had more opportunities to reach others in their communities:

“My role was that I formed a team of students, LRBT [eye care provider] had given them a training and those students who had a visual problem in the school … were identified. I was monitoring the team. … Furthermore, these students also performed testing outside … the school, in the town.” [**Female teacher, government school]**

All the three optometrists interviewed agreed that in some schools, students had been given a basic screening training. However, they did not think it was an effective approach and believed that this could also contribute to inaccurate screening:

“They were fifty-fifty. I mean some of them [teachers] were working very sincerely and seriously. But there are some teachers who have been trained but they asked their students to do the … testing ... So, some teachers were not serious”. [**Optometrist 1**]

### Making referrals

The teachers were provided with information about the referral process and were given referral forms during the training. The teachers were instructed to indicate on the forms whether the student passed or failed the test separately for the left eye and for the right eye. The filled forms were used to refer students to an optometrist for an examination, diagnosis, and management. Data from the interviews shows that not all teachers understood the purpose of the referral forms and how to complete them. Five teachers did not know that the results had to be recorded separately for each eye. The incorrectly completed referral forms resulted in extra work for the optometrists, who had to check and correct the forms.

“Yes, there is one more issue, with the form. We made three columns on the form, the right eye, the left eye, and other problems. So, the teachers … mixed up these columns and marked them ‘fail’ in all the columns. So, we … took much time to identify the problem [using these forms] and a lot of our time was wasted … ”. [**Optometrist 3**]

#### Post-training support for teachers

The trainers reported that they had provided post-training support to the teachers by sharing their contact details and asking the teachers to contact them for any questions or clarifications. Twelve teachers however said that they did not have the optometrists’ contact details; and those who did, had not contacted the optometrists for any advice. The teachers said that they wanted the optometrists to visit them once a month to monitor and discuss the screening. The optometrists pointed out that the supervisory visits to the schools were agreed within the project but only 6 months after the training. Teachers believed that 6 months was a lengthy period to wait, as one teacher explained:

“It is working fine but as I said earlier, it would be good to have them [optometrists] revisit once a month if only for 15 minutes. They are visiting after 6 months. This should not happen. They should visit at least once a month. This is a matter of eyes. There could be problems. The hospital is close to us.” [**Male teacher, private school]**

#### Teachers’ motivation and commitment

Both the teachers and the optometrists described the strong support they had received from the school administration to conduct the vision screening programme. In addition to nominating the teachers to participate in the programme, the schools allowed time for the students to take a test and further examination and provided transport to those, who needed to visit hospital. The teachers interviewed also demonstrated a good understanding of the importance of vision; all were positive about the programme and its benefits.

The participants pointed out that the programme was particularly important for the children from poorer families, who could not visit health facilities for regular check-ups. All participants also appreciated that the programme covered the costs of medicines and eyeglasses provided at the hospitals. The teachers were highly committed to learning about vision testing and appreciated that they themselves received free eye tests and spectacles, as part of the programme.

The majority (10/14) of teachers wanted to continue doing the screening. The improvements they recommended were refresher training and an increased number of supervisory visits by the trainers. One teacher suggested considering a designated role for vision screening in schools. The only concern raised by a number of teachers interviewed was their time. Teachers said that they were busy with their other day to day teaching duties – and conducting vision screening and completing forms was an additional burden, adding more tasks to an already full workload:

“The difficulty was that it is time consuming and we have other tasks to complete at school as well. So yes, that is the issue I faced … [...]...” [**Female teacher, private school]**

## Discussion

This study investigated the factors contributing to inaccurate vision screening performed by teachers in selected schools in Pakistan and specifically, the high number of false positives referred unnecessarily to hospitals for further examination and treatment. We solicited perspectives from the teachers and eye care workers directly involved in the implementation of the vision screening programme [28–30]. Our findings complement other research showing that the quality of vision screening is dependent on the effectiveness of interactions between education and health systems and more specifically, the quality of training and support given to the teachers [7].

Our findings show that the vision screening programme was well understood and appreciated by the teachers and other education staff, but the quality of the programme was undermined by a number of operational issues. For example, the duration of the teacher training programme was significantly reduced due to the discrepancy between the programme targets and resources allocated to achieve them. As a result, the training was rushed; the concepts and procedures were not sufficiently explained, and the teachers did not have adequate time to practice their skills. The impact of insufficient training on teachers’ confidence and the rate of over referrals has been shown in other vision screening studies. Kaur et al. showed that the teachers, who are not fully confident in their skills, are over cautious and over refer in an attempt to avoid missing out any child, where they may have doubts [23].

Our study also shows that the teachers had little support and supervision after the training. Although the contact details of the trainers had been provided, in practice very few teachers contacted the trainers. The supervisory visits were organised only 6 months after the training. By that time, many teachers had lost some of the acquired skills or developed inaccurate and incorrect practices. The study suggests that specific difficulties experienced by the teachers included poor understanding of the distances required for visual screening, the use of the E-chart, confusions about distance vision and near vision screening and misunderstanding of how to complete the referral forms. More frequent and regular supervisions could have mitigated the impact of the reduced duration of the training and could have improved the skills of the teachers. The reasons for the lack of support supervision had more to do with systems-level factors like poor planning, organization, and delivery of the programme rather than an unwillingness or inability on the part of the optometrists (trainers) to provide the appropriate support.

Similarly, a refresher training course could have helped to supplement the learning and address the issues that arose during the screening. In this study, teachers suggested that in the absence of resources to conduct a refresher course, optometrists could have engaged teachers in the rescreening of incorrectly referred students. This way, the optometrists could have guided teachers towards understanding the gaps in their knowledge and developing more accurate screening skills. Similar suggestions have been made in other literature [7, 24].

Our findings also suggest that there was some discrepancy in the understanding of the complexity of the training between the trainers and the trainees. The training content, and materials, did not reflect exactly what the teachers were being asked to do in practice, which contributed to the confusion. This indicates a need for not only better trainers, but also better training materials development consistent with what the teachers will ultimately be asked to do. While the content of the training appeared to be simple and straightforward to the optometrists, it was not appropriately adapted for non-clinical audiences. Evidence suggests that it often happens with the trainers, who may be fully competent in their professional field but may not be experienced as trainers [31, 32]. Future training programmes should place more emphasis on the identification and preparation of the trainers to make sure they can effectively deliver the training to non-health professionals.

Furthermore, the teachers’ personal interests and commitments to vision screening programmes should be considered, when selecting those to be trained. In this study, not all trainees were motivated, some teachers did not take this new responsibility seriously. Evidence from other school-based health programmes shows that the lack of interest and low levels of commitment among the teachers affect their levels of participation in health programmes and ultimately the quality of their work [33–35].

In addition, many teachers participating in this study reported being overburdened with other work, which affected both their levels of participation in the training and the time they gave to the screening. Although teachers understood and appreciated the benefits of the programme, additional tasks associated with the screening affected their motivation and commitment. Some teachers delegated screening to the students as they did not have sufficient time in their day-to-day work schedules, some did not strictly follow the procedures; some did not pay attention to the correct recording of the results. Delegating vision screening to students seemed supported by the ophthalmic team of trainers who also acknowledged training students in some schools. The overburden of teachers with additional (health-related) tasks and resulting errors and inaccuracies has been reported in other studies [7]. In India, the increased workload among the teachers affected the effectiveness of vision screening [36]. In Nigeria, the overburden of the personnel involved in deworming programmes affected the morale and the relationships between the staff within the schools [30]. The vision testing guidelines are also crucial for accuracy of the test [37] and although many teachers spoke about having the guidelines, we did not have strong evidence that the teachers were using them regularly. That some of the teachers could not immediately locate or remember where the guidelines had been kept underscores the need to develop appropriate strategies for regular support and mentorship from eye care professionals. The findings corroborate the results from other studies, where lack of confidence and insufficient attention given to the use of protocols resulted in the laxity of the procedures and poor results [25].

There are a number of limitations in this study. First, all the findings are based on self-reported interviews with a small sample of study participants. We did not observe the programme design and monitoring activities, training, or the screening procedures applied in practice. Second, our selection of the study schools was purposive, based on the high rates of false positives, and the individual teachers were selected based on their availability and willingness to be interviewed. It means that we do not have data from the better performing schools and cannot definitively say whether the issues identified in this study were indeed the drivers of poor performance. In addition, we cannot say whether the teachers, who agreed to be interviewed were indeed the teachers with the highest numbers of inappropriate referrals. It is also important to note that this study had only information on false positives referred to the optometry teams. We did not carry out tests to verify the results of those children who were categorised as having normal vision. We therefore cannot estimate the number of false negatives and cannot fully assess the accuracy of the screening in this programme. Additionally, we did not assess the commitment level of the teachers and we do not have detailed data on teachers’ feelings or perspectives with regard to their confidence in screening children’s eyes. Therefore our analysis on whether they may have been worried that they might miss something and thus end up being over cautious and over-referring is limited [23]. Finally, this study was cross-sectional, providing data at only one point in time. The study design was unable to ascertain whether teachers trained at different points throughout the programme applied their skills differently.

## Conclusion

Although the school-based vision programme was well received and supported by the schools and eye care providers, there were a number of issues that undermined the quality of vision screening. These included the limited time allocated to the training; limited supervision and support; insufficient use of guidelines and screening protocols and the heavy workload of the teachers. Our analysis suggests that for school-based vision screening programmes to be provided on a larger scale in future, supportive supervisory structures should be put in place to ensure that teachers receive an adequate level of support and their routine teaching workload is carefully considered to maximise effectiveness and efficiency.

## Supplementary Information

**Additional file 1.**

**Additional file 2.**

**Additional file 3.**

## Data Availability

The datasets analysed (transcripts) during the current study are not publicly available since they include personal details but could be made available in anonymized form from the corresponding author on reasonable request.

## Abbreviations

LRBT: Layton Rahmatulla Benevolent Trust
URE: Uncorrected Refractive Errors
LMICs: Low- and Middle-Income Countries
REC: Research Ethical Committee
