# Supplementary material for: School-based vision screening in Quetta, Pakistan: a qualitative study of experiences of teachers and eye care providers
Source: BMC Public Health. 2021 Feb 16;21:364. doi: 10.1186/s12889-021-10404-9 (PMC7885518; doi:10.1186/s12889-021-10404-9)
Supplement: Supplementary file 1 — Additional file 1. [file 12889_2021_10404_MOESM1_ESM.docx]

| **Open-Ended Questions Used During Interviews with Teachers** | |
| --- | --- |
| 1. | What is your role in the visual acuity testing programme? |
| 2. | How long have you been testing visual acuity? |
| 3. | What skills do you think are required to test visual acuity? |
| 4. | Do you think you possess these skills? Why do you think this? |
| 5. | What training have you received on testing visual acuity? |
| 6. | Do you get any other support around this area of your work? |
| 7. | Who trained you? Were all your questions addressed by the trainer? |
| 8. | Do you have any reference materials you use to remind you about the procedures?  Please tell me about the visual acuity testing procedure. |
| 9. | What examination tools have been provided for the visual acuity testing? Do you have all these tools? If not, do you improvise and how? |
| 10. | Where do you carry out the visual acuity testing? Where is ideal to do so? |
| 11. | What is the correct testing distance? |
| 12. | How do you know a student had distant vision? What are the diagnostic features? |
| 13. | How do you know a student has near vision? What are the diagnostic features? |
| 14. | How do you record the results of the test? |
| 15. | What do you do when you are not sure of the diagnosis? |
| 16. | What happens to the children you identify as having an eye problem? How do you make a referral to the optometrist? |
| 17. | What do you do if you identify a child with a problem, but they don’t get glasses? |
| 18. | How often do you do the visual acuity tests? |
| 19. | Do you find challenges in conducting the tests with any particular groups of students? (e.g., girls, boys, younger, older, other distinguishing features…) |
| 20. | Are there any other challenges you find with the visual acuity testing we have not already discussed? |
| 21. | How important is it for refractive error to be corrected in children of school going age? |
| 22. | How does the visual acuity testing fit in with your teaching responsibilities? |
| 23. | How does the school management support the visual acuity testing programme? |
| 24. | What organisations outside of the school are involved in the programme and what are their roles? |
| 25. | Does the program work well? If not, what can be done to improve it? |
