# Supplementary material for: School-based vision screening in Quetta, Pakistan: a qualitative study of experiences of teachers and eye care providers
Source: BMC Public Health. 2021 Feb 16;21:364. doi: 10.1186/s12889-021-10404-9 (PMC7885518; doi:10.1186/s12889-021-10404-9)
Supplement: Supplementary file 2 — Additional file 2. [file 12889_2021_10404_MOESM2_ESM.docx]

| **Open-Ended Questions Used During Interviews with Optometrists** | |
| --- | --- |
| 1. | Please tell me about your role in the school-based visual acuity testing programme. |
| 2. | From your perspective, what is the objective of the programme? |
| 3. | How were you selected to be a trainer? |
| 4. | How are teachers who participate in the program selected? |
| 5. | What does the training entail? What does it cover? |
| 6. | What are the main challenges you encounter in providing the training? |
| 7. | Does every teacher who participates go on to test visual acuity or do some not ‘pass’ the training? |
| 8. | Is there a refresher training? If not, in your opinion, would this be useful? |
| 9. | Is there any support to the teachers carrying out the visual acuity testing beyond the training? e.g., supervision? |
| 10. | What equipment / facilities do teachers need to conduct the testing? Do they have access to these? |
| 11. | In your opinion what are the main challenges of the programme? |
| 12. | In your opinion, what are the main benefits of the programme? |
| 13. | What are the mistakes that can happen in teacher led visual acuity testing? |
| 14. | In your opinion, why do false positives occur? What is the impact of false positives? |
| 15. | How can false positives be reduced? |
| 16. | How do you find out a mistake has been made by a teacher? |
| 17. | How do you tell the students/ their parents that a mistake was made? |
| 18. | If a student has a more complex ophthalmic problem, what steps do you take? |
| 19. | What records/ data do you keep about the school based visual acuity testing programme? Who do you report to on the programme activities? |
| 20. | Do you give feedback to teachers about false positives? What kind of feedback do you give? And how do you address the issues? |
