# Supplementary material for: School-based vision screening in Quetta, Pakistan: a qualitative study of experiences of teachers and eye care providers
Source: BMC Public Health. 2021 Feb 16;21:364. doi: 10.1186/s12889-021-10404-9 (PMC7885518; doi:10.1186/s12889-021-10404-9)
Supplement: Supplementary file 3 — Additional file 3. [file 12889_2021_10404_MOESM3_ESM.docx]

| **Content and Information covered in the Guidelines and Leaflets** | |
| --- | --- |
| 1. | What is vision screening and why is it needed in school and community-based programmes |
| 2. | What is needed for vision testing (E card, Measuring tape, record sheet and referral sheet) in a well-lit space |
| 3. | Which side of the card is for what purpose? D and N explanation |
| 4. | What are the important points to consider for vision testing with children? (in this section the exact distance to measure, how to explain to the child regarding E chart legs pointing in which direction, what to do if the child already wears glasses, the space should be well-lit and well ventilated where vision is being tested, etc. etc.) |
| 5. | Detailed step by step directions for checking Distant Vision. |
| 6. | Detailed step by step directions for checking Near Vision |
| 7. | Ten key messages from WHO regarding child eye health and common eye diseases in children with pictorial explanation |
|  |  |
